# Is There Utility in Transvaginal Cervical Length Surveillance After Cerclage Placement for the Prediction of Spontaneous Preterm Birth?

**DOI:** 10.7759/cureus.64818

**Published:** 2024-07-18

**Authors:** Elizabeth Cochrane, Chloe Getrajdman, Nicola F Tavella, Ana Capi, Tahera Doctor, Manasa G Rao, Elianna Kaplowitz, Guillaume Stoffels, Joanne Stone, Noel Strong, Chelsea DeBolt

**Affiliations:** 1 Obstetrics and Gynecology, Icahn School of Medicine at Mount Sinai, New York, USA; 2 Obstetrics and Gynecology, Columbia University College of Physicians and Surgeons, New York, USA; 3 Biostatistics, Icahn School of Medicine at Mount Sinai, New York, USA

**Keywords:** transvaginal ultrasound, short cervical length, ptb: preterm birth, cervical cerclage, transvaginal ultrasonography cervical length

## Abstract

Introduction: Transvaginal cervical length (TVCL) surveillance post-transvaginal cerclage placement is not universally performed, despite the correlated risk of short TVCL with spontaneous preterm birth (sPTB). This study evaluated if patients with a TVCL <2.5 cm after cerclage placement had higher odds of sPTB than those with a TVCL ≥2.5 cm after cerclage placement.

Methods: This retrospective cohort study included patients with a singleton, non-anomalous gestation with a transvaginal cerclage who had TVCL surveillance post-cerclage placement. The primary outcome was the odds of sPTB among patients with TVCL <2.5 cm vs TVCL ≥2.5 cm after cerclage placement. Transvaginal cerclage placement indications included history indicated, physical exam indicated, and ultrasound indicated. Outcomes were assessed using univariate and multivariate analysis while adjusting for progesterone use, TVCL before cerclage placement, and cerclage indication.

Results: The analysis included 210 patients, and the sPTB rate was 46.7%. Those with sPTB underwent cerclage placement at later gestational ages, had higher rates of exam-indicated cerclage, and were more likely to be prescribed vaginal progesterone. Patients with a TVCL of <2.5 cm after cerclage placement did not have significantly increased odds of sPTB (OR: 2.8, 95% CI: 0.9-8.7, p=0.07); however, patients with a TVCL <2.0 cm had significantly increased odds of sPTB (OR: 6.3, 95% CI: 2.2-18.8, p<0.001).

Conclusion: In patients with transvaginal cerclage, there does not appear to be increased odds of sPTB with TVCL <2.5 cm after cerclage placement; however, there does appear to be an increased odds of sPTB in patients with a TVCL of <2.0 cm after cerclage placement.

## Introduction

Preterm birth (PTB), defined as birth between 20 weeks 0 days and 36 weeks six days gestational age, remains a significant obstetrical issue in the United States (US) [1-3]. There have been many advances in the prevention of PTB, including progesterone use (intravaginally and intramuscularly), transvaginal cervical length evaluation, and the use of cervical cerclages; however, PTB continues to be a significant contributor to neonatal morbidity, neonatal mortality, and healthcare costs [2,3]. Additionally, as intramuscular 17-alpha hydroxyprogesterone caproate (17-OHPC), previously a cornerstone of PTB intervention, was recently withdrawn from the US market and is no longer recommended for the use of prevention of PTB in patients with a history of PTB, new and updated research is needed to understand and explore alternative managements for the prevention of PTB [4,5].

While transvaginal cervical length (TVCL) assessment is performed in patients at high risk for PTB before 24 weeks gestation, current guidelines do not recommend ultrasound assessment of TVCL after cerclage placement [2,3]. This study aimed to evaluate if short TVCL after cerclage placement is associated with increased odds of spontaneous PTB (sPTB). We hypothesized that the odds of sPTB among patients with a TVCL <2.5 cm after cerclage placement would be greater than those with a TVCL ≥2.5 cm after cerclage placement.

This article was previously presented as an abstract at the Society of Maternal-Fetal Medicine 43rd Annual Pregnancy Meeting on February 10, 2023.

## Materials and methods

This retrospective cohort study was performed at a single urban academic center in the US. This study was approved by the medical center's institutional review board (IRB) on 12/16/2021 (IRB #21-01755) with a waiver of consent. At this institution, patients at high risk of preterm birth (for example, those with a short cervix, with a cerclage in place, or a history of preterm birth) undergo TVCL screening every two weeks between 16 and 28 weeks gestation. Patients with a transvaginal cerclage routinely begin TVCL surveillance about one to two weeks after cerclage placement. Patients were included in our study if they had a singleton, non-anomalous gestation with a transvaginal cerclage who had TVCL surveillance post-placement and delivered at our institution. Transvaginal cerclage indications included ultrasound, physical exam, and history indicated transvaginal cerclage. Patients were excluded if prenatal ultrasound imaging was not available or if they had a TVCL indication of a uterine anomaly or a history of cervical surgery. Patients were identified retrospectively by querying our ultrasound imaging software to identify patients with a cerclage and transvaginal cervical length. A chart review assessed patient eligibility and facilitated data collection. The primary outcome was sPTB, defined as either spontaneous preterm labor or preterm premature rupture of membranes with delivery prior to 37 weeks gestation. We also examined the association between sPTB and TVCL measurements at differing post-cerclage placement times.

Demographic data collected included age at delivery, BMI at the start of pregnancy, self-reported race and ethnicity, cerclage indication, progesterone use, cervical length screening indication, nulliparity, and gestational age at cerclage placement. Of note, at the time of patient care included in this retrospective study, 17-OHPC was not yet removed from the market and was routinely being prescribed. Delivery data collected included gestational age at delivery and infant birthweight. 

Study data were collected and managed using Research Electronic Data Capture (REDCap) tools hosted at our institution [6,7]. REDCap is a secure, web-based software platform designed to support data capture for research studies, providing (1) an intuitive interface for validated data capture; (2) audit trails for tracking data manipulation and export procedures; (3) automated export procedures for seamless data downloads to common statistical packages; and (4) procedures for data integration and interoperability with external sources [6,7].

TVCLs were obtained by accredited sonographers and interpreted by maternal-fetal medicine physicians. At the time of this study, our ultrasound unit used Cervical Length Education and Review (CLEAR) guidelines in combination with the American Institute of Ultrasound in Medicine (AIUM) criteria for standardized transvaginal cervical length assessment. These guidelines recommended the following imaging protocol: an empty patient bladder, full visualization of the cervix in the mid-sagittal plane with minimal cervical pressure transvaginally, and magnification of the image so the cervix occupies 75% of the ultrasound screen [8]. Three measurements were obtained from the internal cervical os to the external cervical os, and the shortest and best cervical length measurement was used and reported [8]. 

Demographic characteristics were compared between groups using T-tests or Wilcoxon rank sum tests for continuous measures and Chi-square or Fisher’s exact tests for categorical measures, as appropriate. The association between sPTB and TVCL was first assessed based on the accepted definition of a short cervix as <2.5 cm. We then assessed a cervical length cutoff of <2.0 cm and various other cervical length distances and changes. Univariable and multivariable logistic regression was used to examine the association between TVCL measurement and sPTB. Results were considered statistically significant at the p<0.05 level of significance. All analyses were performed using SAS version 9.4 (SAS Institute, Cary, NC).

## Results

Two hundred thirty-seven patients were identified. Eight patients were excluded for not having TVCL surveillance after cerclage placement, seven patients were excluded for having a medically indicated preterm birth, and 12 patients were excluded for having an indication for TVCL surveillance of either “uterine anomaly” or “history of cervical surgery,” leaving 210 patients in the final cohort. Among the 210 patients in the final analysis, sPTB rate was 46.7%. Those with sPTB underwent cerclage placement at later gestational ages, had higher rates of physical exam-indicated cerclage, were more likely to be prescribed vaginal progesterone and less likely to be prescribed intramuscular progesterone (Table 1). Additionally, patients who had sPTB were less likely to be non-Hispanic Black, had higher rates of PPROM and intraamniotic infection, and were less likely to have hypertensive disorders of pregnancy (Table 1). 

**Table 1:** Patient demographics. SD: standard deviation; IQR: interquartile range; TVUS: transvaginal ultrasound; GA: gestational age; CL: cervical length; PPROM: preterm premature rupture of membranes; L&D: labor and delivery. +Chi-square test for association and Fisher’s Exact test was used for categorical variables. T-test or Wilcoxon rank sum test was used for continuous variables. Four patients in the preterm birth group are missing infant birthweight in grams data. *These variable levels (e.g., “not given”, “not reported”, “other” and/or “unknown”) are removed from p-value calculations. **Categories are not mutually exclusive.

	All (n=210)	Spontaneous preterm birth (n=98)	Full-term birth (n=112)	p-value^ +^
	Mean (SD)	Mean (SD)	Mean (SD)	
Age at delivery (years)	32.0 (5.5)	31.7 (6.6)	32.2 (4.4)	0.60
BMI at start of pregnancy (kg/m^2^)	29.7 (6.1)	28.9 (5.9)	30.5 (6.2)	0.07
	Median (IQR)	Median (IQR)	Median (IQR)	
Cerclage to TVUS (days)	10 (7, 13)	9.5 (7, 13)	10 (7, 14.5)	0.52
GA at cerclage placement (weeks)	17 (14, 21)	19 (14, 21)	15 (14, 20)	0.01
GA at delivery (weeks)	37 (33, 38)	32.5 (28, 35)	38 (37, 39)	<0.0001
Infant birthweight (grams)	2890 (2070, 3250)	2007.5 (1180, 2460)	3205 (2967.5, 3445)	<0.0001
	No. (%)	No. (%)	No. (%)	
Race/ethnicity				0.02
Non-Hispanic Black	83 (39.5)	29 (29.6)	54 (48.2)	
Non-Hispanic Asian	18 (8.6)	13 (13.3)	5 (4.5)	
Non-Hispanic White	14 (6.7)	6 (6.1)	8 (7.1)	
Hispanic	24 (11.4)	15 (15.3)	9 (8.0)	
Not given*	5 (2.4)	3 (3.1)	2 (1.8)	
Non-Hispanic other	64 (30.5)	32 (32.7)	32 (28.6)	
Unknown*	2 (1.0)	0 (0)	2 (1.8)	
Nulliparous	63 (30.0)	31 (31.6)	32 (28.6)	0.63
Spontaneous labor	160 (76.2)	98 (100.0)	62 (55.4)	<0.0001
Pre-existing medical conditions**				
Type 2 diabetes mellitus	7 (3.3)	5 (5.1)	2 (1.8)	0.26
Chronic hypertension	21 (10.0)	10 (10.2)	11 (9.8)	>0.99
Pregnancy issues**				
PPROM	61 (29.0)	61 (62.2)	0 (0)	<0.0001
Intra-amniotic infection	24 (11.4)	19 (19.4)	5 (4.5)	<0.001
Pre-eclampsia with severe features	8 (3.8)	3 (3.1)	5 (4.5)	0.73
Pre-eclampsia without severe features	3 (1.4)	1 (1.0)	2 (1.8)	>0.99
Gestational hypertension	7 (3.3)	0 (0)	7 (6.3)	0.02
Hypertensive disorder of pregnancy	18 (8.6)	4 (4.1)	14 (12.5)	0.03
Gestational diabetes (GDMA1 + GDMA2)	22 (10.5)	8 (8.2)	14 (12.5)	0.31
Reason why TVCL was initiated				
History of preterm birth ≥20 weeks	112 (53.3)	45 (45.9)	67 (59.8)	0.04
TVCL <2.5 cm <24 weeks	74 (35.2)	45 (45.9)	29 (25.9)	<0.01
History of second-trimester loss <20 weeks	46 (21.9)	15 (15.3)	31 (27.7)	0.03
Cerclage indication				<0.0001
History indicated	106 (50.5)	36 (36.7)	70 (62.5)	
Ultrasound indicated	50 (23.8)	23 (23.5)	27 (24.1)	
Physical exam indicated	46 (21.9)	35 (35.7)	11 (9.8)	
Other	8 (3.8)	4 (4.1)	4 (3.6)	
Progesterone**				
Any	182 (86.7)	85 (86.7)	97 (86.6)	0.98
Vaginal progesterone	105 (50.0)	59 (60.2)	46 (41.1)	<0.01
Intramuscular progesterone	90 (42.9)	34 (34.7)	56 (50.0)	0.03

When TVCL was examined as a categorical variable and adjusted for corresponding shortest TVCL before cerclage, progesterone use, and indication for cerclage, patients with a TVCL of <2.5 cm after cerclage placement did not have significantly increased odds of sPTB (OR: 2.8, 95% CI: 0.9-8.7, p=0.07, Table 2). However, patients with a TVCL <2.0 cm at any gestational age after cerclage placement (OR: 6.3, 95% CI: 2.2-18.8, p<0.001), and specifically between 20- and 24-weeks gestation (OR: 8.0, 95% CI: 2.2-28.3, p<0.01), had increased odds of sPTB (Table 2).

**Table 2:** Odds of spontaneous preterm birth by TVCL. n: number; Obs: observed; TVCL: transvaginal cervical length. +All models are logistic regression models adjusted for corresponding shortest CL before cerclage, progesterone use (yes/no), and indication for cerclage (history, ultrasound, physical exam, other indication). *Significance is calculated at the p<0.01 level after Bonferroni correction for multiple comparisons.

	Spontaneous preterm birth (n=98)	Full-term birth (n=112)	Adjusted odd ratio (95% CI)^ +^	p-value^+^*
	n/n Obs. (%)	n/n Obs. (%)		
All patients				
Shortest TVCL after cerclage <2.5 cm	82/98 (83.7)	50/112 (44.6)	2.8 (0.9-8.7)	0.07
Shortest TVCL after cerclage <2 cm	73/98 (74.5)	31/112 (27.7)	6.3 (2.2-18.8)	<0.001
Shortest TVCL after cerclage 20-23 weeks six days <2.5 cm	64/90 (71.1)	34/98 (34.7)	2.7 (1.0-7.5)	0.05
Shortest TVCL after cerclage 20-23 weeks six days <2 cm	48/90 (53.3)	21/98 (21.4)	8.0 (2.2-28.3)	<0.01

## Discussion

This study found that there does not appear to be increased odds of sPTB with TVCL <2.5 cm after cerclage placement; however, there is an increased odds of sPTB in patients with a TVCL of <2.0 cm after cerclage placement. Prior studies have shown that transvaginal cerclages lengthen the cervix and decrease the risk of preterm birth [2,3,9]. However, patients with a cerclage remain at an increased risk of preterm birth, and this study worked to help delineate those patients at greater risk. While there are multiple studies assessing cervical length in patients without a cerclage, few studies have assessed cervical lengths and odds of spontaneous preterm birth in patients with cervical cerclages, particularly at 20-24 weeks gestation. Additionally, studies have examined the effect of the location of the cervical cerclage within the cervix [10,11]. Battarbee et al. worked to identify ultrasonographic criteria associated with an increased risk of PTB in patients with a cervical cerclage [10]. Among them was a short cervical length between the cerclage and external os [10]. Additionally, Ridout et al. assessed the longitudinal change in cervical length post-cervical cerclage placement in patients with a history of a failed vaginal cerclage, and they found that in their cohort, cervical length was a good predictor of sPTB [11]. These studies are of particular interest as our present study suggests that patients with sPTB in our cohort had overall shorter TVCL.

While PTB rates are increasing in the United States, it is anticipated that transvaginal cervical cerclage in pregnancy will remain a significant and prevalent intervention [1,3]. Dijkstra et al. (2000) found that progressive shortening of the cervix between 24- and 32-weeks’ gestation was associated with an increased risk of PTB [12]. While our study focused on the odds of sPTB with a TVCL <2.5cm at 20-24 weeks gestation and any gestational age after cerclage placement, future studies can focus on the rate of cervical change as Dijkstra et al. did [12]. Prospective studies to assess cervical length post-cerclage placement are needed. Moreover, future studies may focus on randomized controlled trials to help identify those patients with cervical cerclage in situ who are at an increased risk of preterm delivery. Baxter et al. in 2005 found that a reinforcing cerclage in patients <24 weeks with a short cervix (defined as <2.5 cm) with a history of indicated cerclage already in situ led to increased rates of preterm birth compared to those patients who had continued expectant management [13]. While their study used a threshold of 2.5 cm, they did not assess the PTB outcomes in patients with a TVCL <2.5 cm post-cerclage and ≥2.5 cm without an additional intervention [13]. Additionally, our study suggested increased odds of sPTB in patients with a TVCL <2.0 cm post-cerclage, not <2.5 cm. Identifying the clinical characteristics of these patients may help time fetal interventions such as betamethasone administration, magnesium sulfate administration, and maternal interventions, although further research is needed. Additionally, future research is required to determine if different TVCL thresholds for a “short” cervix exist in patients with a transvaginal cervical cerclage. 

Strengths and limitations

As our study is retrospective, there are possible limitations of errors in data collection, electronic medical record documentation, and confounding variables of preterm birth that were not collected or for which we did not account. Additionally, three indications for transvaginal cerclage were included in this study (history, ultrasound, and physical exam), as there were higher rates of sPTB in the physical exam indicated group, there may be confounding effects that were unable to be fully appreciated in this study. As patients were identified using a query in our ultrasound imaging software, patients who did not have proper documentation in their ultrasound reports were likely missed. Furthermore, ultrasound imaging is subject to both inter-operator and inter-interpreter variability between cervical length measurements. Strengths of this study include a diverse patient population and a relatively large sample size for a relatively rare cohort of pregnant patients, which allows for generalizability. Additionally, all cervical lengths used in this study were obtained transvaginally, which, as opposed to transabdominal, is more reproducible and is not affected by fetal positioning and maternal factors such as obesity [3]. 

## Conclusions

In patients with transvaginal cerclage, there does not appear to be increased odds of sPTB with TVCL <2.5 cm after cerclage placement. However, there does appear to be an increased odds of sPTB in patients with a TVCL of <2.0 cm. Future research is needed to determine the utility of TVCL surveillance after cerclage placement and if alternative cervical length thresholds should be considered.
